# BIOPSYCHOSOCIAL DETERMINANTS OF COGNITIVE FUNCTION AMONG BLACK OLDER ADULTS

**DOI:** 10.1093/geroni/igad104.0508

**Published:** 2023-12-21

**Authors:** Amy Thierry

**Affiliations:** Xavier University of Louisiana, New Orleans, Louisiana, United States

## Abstract

Black older adults in the United States have twice the risk of being diagnosed with Alzheimer’s disease and related dementias than their white counterparts. However, there remains a gap in knowledge of which factors may contribute to this disparity as well as mechanisms that either increase or decrease risk for poor cognitive outcomes, specifically in older Black adults. This presentation will highlight recent interdisciplinary population health research on the biopsychosocial determinants of cognitive function among Black adults 65 years and older in the Health and Retirement Study. Using within-group regression analyses, studies examine associations between multiple measures across neighborhood, psychosocial, and biomarker domains with cognitive function, measured by Telephone Interview for Cognitive Status scores (range: 0-35) which captures memory and overall mental status. We find that high neighborhood disorder and low social cohesion were associated with worse cognitive function, especially for more highly educated Black older adults and those living in urban communities. Greater frequency of experiences of everyday discrimination among Black older adults was also related to lower cognitive function. Volunteering and having a high sense of purpose in life were associated with better cognitive function for older Black men and women, while being physical active was associated with increased cognitive function for older Black women. C-reactive protein, a measure of systemic inflammation, was positively associated with cognitive function. Overall, this body of work demonstrates the need to further critically assess both risk and resilience processes underlying cognitive function among older Black adults towards reducing current cognitive health inequities.

